# Admission rates and clinical profiles of children and youth with eating disorders treated as inpatients before and during the COVID-19 pandemic in a German university hospital

**DOI:** 10.3389/fpubh.2023.1281363

**Published:** 2023-11-30

**Authors:** Ann-Sophie Silber, Simeon Platte, Afsheen Kumar, Sukhdeep Arora, Dennis Kadioglu, Marvin Schmidt, Holger Storf, Andreas G. Chiocchetti, Christine M. Freitag

**Affiliations:** ^1^Department of Psychiatry, Psychosomatics and Psychotherapy of Childhood and Adolescence, Goethe University Frankfurt, University Hospital, Frankfurt am Main, Germany; ^2^Institute of Medical Informatics (IMI), Goethe University Frankfurt, University Hospital, Frankfurt am Main, Germany

**Keywords:** anorexia nervosa, COVID-19 pandemic, child, adolescent, youth

## Abstract

**Introduction:**

Children and youth at risk for mental health disorders, such as eating disorders (ED), were particularly affected by the COVID-19 pandemic, yet evidence for the most seriously affected and thus hospitalized youth in Germany is scarce.

**Methods:**

This crosssectional study investigated anonymized routine hospital data (demographic information, diagnoses, treatment modalities) of patients admitted (*n* = 2,849) to the Department of Child and Adolescence Psychiatry, Psychosomatics and Psychotherapy (DCAPPP) of a German University Hospital between 01/2016 and 02/2022. Absolute and relative number of inpatients with or without ED prior to (01/2016–02/2020) and during the COVID-19 pandemic (03/2020–02/2022) were compared. The effect of school closures as part of social lockdown measures for COVID-19 mitigation on inpatient admission rate was explored as it has been discussed as a potential risk factor for mental health problems in youth.

**Results:**

During the COVID-19 pandemic, ED inpatient admission rate increased from 10.5 to 16.7%, primarily driven by Anorexia Nervosa (AN). In contrast to previous reports, we found no change in somatic and mental disorder comorbidity, age or sexratio for hospitalized youth with ED. However, we did observe a shortened length of hospital stay (LOS) for hospitalized youth with and without ED. In addition, non-ED admissions presented with an increased number of mental disorder comorbidities. In contrast to our hypothesis, school closures were not related to the observed increase in ED.

**Discussion:**

In summary, the COVID-19 pandemic was associated with an increased rate of inpatient treatment for youth suffering from AN, and of youth affected by multiple mental disorders. Accordingly, we assume that inpatient admission was prioritized for individuals with a higher burden of disease during the COVID-19 pandemic. Our findings pinpoint the need for adequate inpatient mental health treatment capacities during environmental crises, and a further strengthening of child and adolescence psychiatry services in Germany.

## 1 Introduction

The COVID-19 pandemic, declared by the Word Health Organization on March 11th, 2020, and associated public health strategies of mitigation, such as lockdown orders and school closures, have been proposed as negative environmental factors on the mental health of children and youth (1–3), with long-term consequences still surfacing. Cumulating studies indicate a corresponding increase in depressive and anxious symptoms (4, 5), a rise in emergency consultations due to mental health problems (6), and a reduction in perceived quality of life (7, 8) in children and youth. Next to the impacts on physical health and changes in work and family life, proposed mediators especially affecting children and youth include the disruption of daily routines, loneliness due to social distancing orders, increased use of virtual communication and social media, as well as reduced availability of extracurricular activities (8, 9).

ED, and especially AN, often lead to extensive medical and mental health consequences, thus constituting a profound individual and public health burden. AN carries a high risk for cachexia-induced organ failure, repetitive hospitalizations, a more than six-fold mortality rate, and increased suicide risk. The recovery rate is less than 50%, indicating a high risk for chronicity, leading to a high personal disease burden and related impairment (10, 11). Meta-analyses (12, 13) and systematic reviews (14–16) reported a rise in ED incidence during the COVID-19 pandemic along with increases in ED symptom severity and comorbid mental disorders. Still, only a small portion of the included studies reported data on children and youth. Restrictive ED regularly emerge in early adolescence, with an onset in childhood resulting in an even poorer prognosis (17, 18). This underlines the necessity for further research on ED in children and youth in the context of the COVID-19 pandemic. Local studies found increases in pediatric hospital admissions for ED in Canada (19–21), North America (22–24), Australia (25), Israel (26), Italy (27, 28), Norway (29), and the Netherlands (30) following the onset of the COVID-19 pandemic. Findings concerning symptom severity or clinical characteristics of children and youth admitted for ED treatment during the COVID-19 pandemic have been diverse: North-American, Canadian and Italian pediatric departments at tertiary care hospitals with special ED-treatment programs registered more critical somato-medical problems (19, 20, 23, 24, 28), whereas similar medical facilities in Australia (25) and Israel (26) found clinical characteristics to be unchanged to pre-COVID-19 samples. Shortened length of stay (LOS) was found at pediatric hospitals in Israel and Italy (26, 27), yet prolonged LOS was observed at a large pediatric hospital with a specialized ED-treatment program in North America and at some Departments of Child and Adolescent Psychiatry in European Countries (31). Most of the few available studies found an increase in co-occurring mental health disorders in children and youth with ED (28, 32, 33). German studies investigating the impact of the COVID-19 pandemic on ED, and especially AN, in children and youth are scarce, but mostly concur with international data, indicating an increase of youth hospitalized for ED during the COVID-19 pandemic (18, 34, 35). The COVID-19 pandemic thus seems to be an environmental risk factor in the Western world, influencing the development and exacerbation of ED in adults and probably children and youth, as well. However, it remains unclear which definitive factors drove this development, especially in the vulnerable adolescent age group, and how it evolved longitudinally during the COVID-19 pandemic.

The primary objective of this study is to retrospectively analyze admission rates of children and youth with ED in a German, university-hospital based DCAPPP. Building on previous research, we specifically tested the hypothesis that the COVID-19 pandemic increased the relative admission rates of children and youth with ED and especially AN. We further tested if the increase was accompanied by a younger average age, a higher rate of co-occurring somatic and mental health disorders including depressive and anxiety disorders, a prolonged LOS and/or a higher rate of early re-admissions, all possibly indicating a higher burden of disease. Additionally, we explored the longitudinal correlation between local school closures as part of German lockdown orders and ED admission rates. This study thus also addresses the paucity of research on the effect of school closures on youth with ED during the COVID-19 pandemic in Germany.

## 2 Materials and methods

The ethical conduct of this study is based on the current version of the Declaration of Helsinki. The medical faculty’s Ethics Board of Goethe University Frankfurt granted ethics approval for this investigation of anonymized routine hospital data (13/06/2022, number 2022–804). Due to anonymization and aggregate analysis of routinely collected hospital data (as stated below), patient consent was not required for this study. The university-hospital’s department of data protection granted the respective data protection approval (31/08/2022).

### 2.1 Study population, data source, and variables

This retrospective hospital-based, cross-sectional study was conducted at the Department of Child and Adolescent Psychiatry, Psychosomatics and Psychotherapy of the University Hospital Frankfurt, Goethe University, Frankfurt am Main, Germany. We investigated children and youth aged 5;0 to 17;11 years hospitalized for emergency or elective psychiatric treatment between January 2016 and February 2022. Our DCAPPP serves all children and youth resident in Frankfurt for emergent and elective inpatient mental health care as well as children and youth living close to Frankfurt for elective mental health care, thus serving around 150.000 children and youth. Retrospective data on the study population was obtained from respective electronic medical records (software: Orbis by Dedalus) including sociodemographic information, routine health care information, diagnoses and treatment modalities, all routinely gathered throughout inpatient treatment, originally for the purpose of health insurance reimbursement. With the core data set covering inpatients only, outpatients in ambulatory treatment at our DCAPPP could not be analyzed in this study. All data was pseudonymized; patient identification numbers (stable over recurring treatment intervals) and case identification numbers (singular to each treatment interval) were generated, respectively, (patient volume < case volume) to distinguish between a first-time admission and a re-admission. Treatment intervals interrupted by temporary discharges due to mandatory COVID-19 mitigation regulations followed by a consecutive re-admission within 3 weeks were considered cumulatively as a single case. Consequently, 2,849 cases, i.e., treatment intervals, of 2,188 individuals, i.e., patients, were investigated. In total, 61% had female sex assigned at birth, and the average age at admission was 14.1 years (SD 2.7). Relative to the onset of the COVID-19 pandemic, the total study period (01/2016 to 02/2022) was divided into a “pre-pandemic” interval from January 1st, 2016, to February 28th, 2020, and a “pandemic” interval from March 1st, 2020, to February 28th, 2022. By date of admission, patients were either allocated to the pre-pandemic or the pandemic interval, the COVID-19 pandemic serving as the main independent variable.

The data source investigated in this study did not yield information on the details of individual admission indications other than the diagnoses. Admission decisions for inpatient treatment at our DCAPPP are based on current German clinical guidelines and the judgment of board-certified child and adolescent psychiatrists or psychotherapists. The intervention of admission itself may serve as a marker for severity of the primary diagnosis and comorbidity load. For ED/AN specifically, respective youth in this study should be considered severly affected. As advised by the German S3 guideline (AWMF), admissions for AN occured in case of rapid weight loss (> 20%) in less than 6 months independent of BMI percentile, servere kachexia (< 3. BMI percentile), insufficient weight restoration during outpatient treatment, insufficient prospect of stabilisation or treatment success in an outpatient setting due to other factors (e.g., comorbidities, problematic family structure, decompensation of family ressources), acute endangerment of self or others (e.g., acute suicidality).

Disorders were diagnosed according to the International Statistical Classification of Diseases, German Modification (“ICD-10”) *via* a team-based, iterative procedure. For each case, the following clinical aspects and treatment modalities served as dependent variables:

- Age at admission (years)
- Sex assigned at birth (% female)
- Length of stay (LOS; number of treatment days from admission to discharge)
- Re-admission (new case, same patient) within 6 months of discharge (% of all admissions)
- First admission (new case, new patient) within 2 years (% of all admissions)
- Total number of all diagnoses at discharge (ICD-10, excluding chapters U, V, X, and Z)
- Number of co-occurring diagnoses regarding mental health (ICD-10, chapter 5: F00-F99: Mental and behavioral disorders) other than the primary diagnosis (total number of mental health diagnoses minus primary diagnosis)
- Number of diagnoses regarding somatic health (ICD-10, chapters other than 5: F00-F99, excluding chapters U, V, X, and Z)
- Proportional case volume (n_cases_, %) of respective diagnostic groups, as specified below

Based on their particular discharge diagnoses, one or more diagnostic groups were assigned to each case:

- ED group: F50 subchapter (i.e., F50.00, F50.01. F50.1, F50.2, F50.3, F50.4, F50.5, F50.8, F50.9) and F98.2/3. Cases lacking such ED-diagnoses were considered within the “non-ED” group.
- ED subgroups: AN (ICD-10: F50.00, F50.01. F50.1), Bulimia Nervosa (BN; ICD-10: F50.2, F50.3), and other ED (ICD-10: F50.4, F50.5, F50.8, F50.9, F98.2, F98.3).
- Depressive Disorders (DD) group: F32.0–9, F33.0–9, F34.1, F34.8/9, F41.2, F48.0, F92.0. Cases lacking any of these diagnoses were classified in the “non-DD” group.
- Anxiety or fear-related Disorders (AD) group: F40.0–9, F41.1–9, F93.0–9, F94.0. Cases lacking any of these diagnoses were classified in the “non-AD” group.

### 2.2 Exploratory investigation of COVID-19 mitigation strategies

For the pandemic interval, changes in non-ED and ED admission rates relative to the pre-pandemic baseline were exploratively correlated with school closures as part of the comprehensive lockdown orders in Germany. We also explored differences in the degree of changes in non-ED and ED admission rates between the two periods of school closures within the pandemic study interval. Duration and timing of school constraints were obtained from (36) and published government directives. School constraints for the average age group in this study were categorized as either (1) schools fully closed (including school holidays, if adjacent or enclosed by a period of school closure) or hybrid teaching (i.e., virtual teaching alternating with in-classroom teaching, or not otherwise specified) OR (2) schools full open.

### 2.3 Statistical analysis

Descriptive statistical analysis was performed with the open-source Software R (version 4.2.2). Dichotomized or categorical variables were summarized by frequencies and percentages (i.e., in reference to the maximum occupancy possible at the respective time interval). Dimensional variables were summarized by means and standard deviation (SD). *p*-values less than 0.05 were considered statistically significant with the null-hypothesis assuming proportional ED admission rates and associated clinical aspects being independent of the COVID-19 pandemic overall and associated school closures in particular. We modeled an average “pre-pandemic baseline” of 1 year on the averaged basis of pre-pandemic admission rates and clinical characteristics, specified per calendar week/month. The respective deviations during the pandemic interval in total and per month of each year were compared by Chi^2^, ANOVA and/or Wilcoxon tests to test whether diagnostic frequencies and clinical aspects as specified above differed significantly between the two study intervals. In general, the available cohort had a power > 0.8 to detect group differences with an effect size d > 0.16 (*t*-test) or w > 0.08 (Chi^2^-test) at an alpha of 0.05 corrected for 39 tests (alpha = 0.0013). A Granger-causality testing was performed to examine the time-lagged causal relationship between school closure severity (schools fully open < hybrid teaching < schools fully closed) and monthly admission rates of AN, allowing for a lag of up to 6 months.

## 3 Results

During the pre-pandemic study interval from January 2016 to February 2020, 1887 hospitalizations (n_cases [pre-pandemic]_) of 1,426 children and adolescents (n_individuals [pre-pandemic]_) were registered with 3.91 ED inpatient admissions per month (198 ED inpatient admissions in total) and 33.34 non-ED inpatient admissions per month (1,689 non-ED inpatient admissions in total), 58.1% with female sex assigned at birth, with an average age at admission of 14.1 years, SD 2.8. During the pandemic study interval from March 2020 to February 2022, inpatient treatment capacities had to be intermittently reduced in compliance with hospital regulations for COVID-19 mitigation. During this interval, 962 hospitalizations (n_cases [pandemic]_) of 762 children and adolescents (n_individuals [pandemic]_ = 749) were registered with 6.64 ED inpatient admissions per month (161 ED inpatient admissions in total) and 33.02 non-ED inpatient admissions per month (801 non-ED inpatient admissions in total), 66.8% of with female sex assigned at birth and an average age at admission of 14.2 years, SD 2.5 (for complete descriptive statistics, see Table 1). Across all cases, independent of diagnostic groups, we found a significant increase in the percentage of female inpatients during the pandemic (58.1% vs. 66.8%, FDR = 3.68 × 10^−5^), a significant decrease in LOS (61.9 days vs. 46.4 days, FDR = 2.51 × 10^−14^), a significant increase in the total number of diagnoses with a significant increase in DD, but not AD (58.9% vs. 69.1%, FDR_DD_ = 1.05 × 10^−6^), and no change in the burden of disease regarding somatic health.

**Table 1:** Statistical analysis of admissions for inpatient treatment to the Frankfurt DCAPPP during the pre-pandemic and pandemic intervals.

	Prepandemic interval	Pandemic interval	*p* value	FDR
Addmissions total	N = 1,887	N = 962		
Individuals total	N = 1,426	N = 762		
Time period (months)	50.66	24.26		
Admissions per month	Npm = 37.25	Npm = 39.65		
Individuals per month	Npm = 28.15	Npm = 31.41		
ED cases	10.49% (N = 198, Npm = 3.91)	16.74% (N = 161, Npm = 6.64)	2.056E-06 ^a^	**1.336E-05**
non-ED cases	89.51% (N = 1,689, Npm = 33.34)	83.26% (N = 801, Npm = 33.02)		
AN	5.09% (N = 96, Npm = 1.89)	9.98% (N = 96, Npm = 3.96)	8.457E-07 ^a^	**6.597E-06**
BN	2.65% (N = 50, Npm = 0.99)	3.12% (N = 30, Npm = 1.24)	4.739E-01 ^a^	7.393E-01
Other ED	2.76% (N = 52, Npm = 1.03)	3.64% (N = 35, Npm = 1.44)	1.955E-01 ^a^	4.012E-01
Age on admission	14.07 (SD = 2.76)	14.23 (SD = 2.52)	1.110E-01 ^b^	2.406E-01
ED cases	14.74 (SD = 2.13)	14.77 (SD = 1.86)	8.954E-01 ^b^	9.799E-01
non-ED cases	13.99 (SD = 2.81)	14.12 (SD = 2.62)	2.403E-01 ^b^	4.468E-01
Female	58.13% (N = 1,097, Npm = 21.65)	66.84% (N = 643, Npm = 26.5)	6.607E-06 ^a^	**3.681E-05**
ED cases	87.88% (N = 174, Npm = 3.43)	88.82% (N = 143, Npm = 5.89)	7.829E-01 ^a^	9.253E-01
non-ED cases	54.65% (N = 923, Npm = 18.22)	62.42% (N = 500, Npm = 20.61)	2.511E-04 ^a^	**1.224E-03**
N diagnoses	3.28 (SD = 1.74)	3.46 (SD = 1.73)	7.560E-03 ^b^	**2.106E-02**
ED cases	3.87 (SD = 1.94)	3.78 (SD = 1.67)	6.096E-01 ^b^	8.358E-01
non-ED cases	3.21 (SD = 1.7)	3.4 (SD = 1.74)	1.023E-02 ^b^	**2.659E-02**
N somatic diagnoses	0.38 (SD = 0.81)	0.36 (SD = 0.73)	4.129E-01 ^b^	7.002E-01
in ED cases	0.50 (SD = 0.93)	0.4 (SD = 0.72)	2.406E-01 ^b^	4.468E-01
in non-ED cases	0.37 (SD = 0.79)	0.35 (SD = 0.73)	5.626E-01 ^b^	8.126E-01
N co-occuring mental health diag.^1^	1.90 (SD = 1.42)	2.1 (SD = 1.49)	3.404E-04 ^b^	**1.475E-03**
in ED cases	2.37 (SD = 1.51)	2.38 (SD = 1.5)	9.744E-01 ^b^	1.000E+00
in non-ED cases	1.84 (SD = 1.4)	2.05 (SD = 1.48)	8.156E-04 ^b^	**3.055E-03**
DD diagnoses	58.93% (N = 1,112, Npm = 21.95)	69.13% (N = 665, Npm = 27.41)	1.082E-07 ^a^	**1.055E-06**
in ED cases	72.73% (N = 144, Npm = 2.84)	72.05% (N = 116, Npm = 4.78)	8.865E-01 ^a^	9.799E-01
in non-ED cases	57.31% (N = 968, Npm = 19.11)	68.54% (N = 549, Npm = 22.63)	8.206E-08 ^a^	**1.055E-06**
AD diagnoses	45.89% (N = 866, Npm = 17.09)	47.92% (N = 461, Npm = 19)	3.049E-01 ^a^	5.404E-01
in ED cases	51.01% (N = 101, Npm = 1.99)	52.8% (N = 85, Npm = 3.5)	7.368E-01 ^a^	9.253E-01
in non-ED cases	45.29% (N = 765, Npm = 15.1)	46.94% (N = 376, Npm = 15.5)	4.407E-01 ^a^	7.162E-01
ED + DD + AD cases	4.56% (N = 86, Npm = 1.7)	7.59% (N = 73, Npm = 3.01)	8.615E-04 ^a^	**3.055E-03**
Length of stay (days)	61.95 (SD = 56.65)	46.41 (SD = 44.17)	1.288E-15 ^b^	**2.512E-14**
ED cases	87.07 (SD = 68.8)	66.8 (SD = 57.21)	2.477E-03 ^b^	**8.050E-03**
non-ED cases	59.01 (SD = 54.32)	42.31 (SD = 39.85)	1.043E-17 ^b^	**4.066E-16**
cases with ED + DD + AD	97.47 (SD = 78.7)	67.59 (SD = 54.03)	5.405E-03 ^b^	**1.622E-02**
W/o previous admission	70.36% (N = 629, Npm = 12.42)	70.06% (N = 674, Npm = 27.78)	9.296E-01 ^b^	9.799E-01
ED cases	58.06% (N = 54, Npm = 1.07)	60.87% (N = 98, Npm = 4.04)	7.592E-01 ^a^	9.253E-01
non-ED cases	71.79% (N = 575, Npm = 11.35)	71.91% (N = 576, Npm = 23.74)	1.000E+00 ^a^	1.000E+00
Readmission within 6 months	39.27% (N = 741, Npm = 14.63)	43.97% (N = 423, Npm = 17.43)	1.577E-02 ^a^	**3.618E-02**
ED cases	42.93% (N = 85, Npm = 1.68)	43.48% (N = 70, Npm = 2.89)	9.169E-01 ^a^	9.799E-01
non-ED cases	38.84% (N = 656, Npm = 12.95)	44.07% (N = 353, Npm = 14.55)	1.303E-02 ^a^	**3.177E-02**
Psychopharmaco. treatment	74.88% (N = 313, Npm = 6.18)	76.54% (N = 372, Npm = 15.33)	5.609E-01 ^a^	8.126E-01
ED cases	70.37% (N = 38, Npm = 0.75)	74.32% (N = 55, Npm = 2.27)	6.215E-01 ^a^	8.358E-01
non-ED cases	75.55% (N = 275, Npm = 5.43)	76.94% (N = 317, Npm = 13.07)	6.493E-01 ^a^	8.440E-01

1: The primary diagnosis, which is always a mental health diagnosis, is not included here (N diag. = N somatic diag. + N co-occuring mental health diag. + 1 primary diag.); a: Chi-Square-test comparing absolute numbers between phases. b: *t*-test comparing average numbers per individual months across phases. diag: diagnosis; N: absolute number; Npm: number per 30 days. SD: standard deviation.

**Figure 1:**
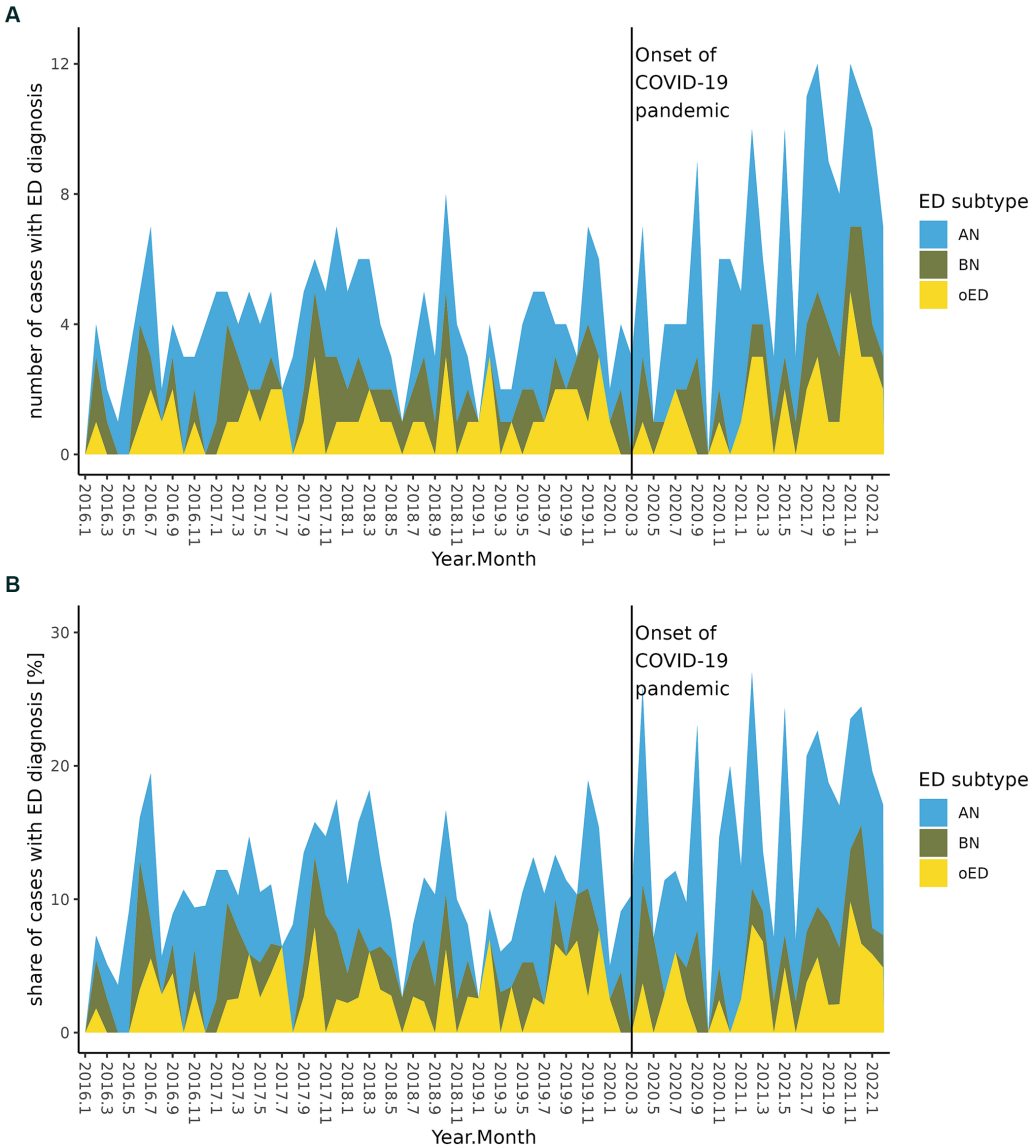
Development of ED inpatient admissions per month, separately depicted by ED subgroup, before and during the COVID-19 pandemic. **(A)** Absolute number of ED inpatient admissions. **(B)** ED inpatient admissions as percentage of all inpatient admissions.

For ED cases, the ratio of treated patients increased significantly from 10.5% during the pre-pandemic interval to 16.7% during the pandemic interval (FDR = 1.34 × 10^−5^). This was mainly driven by patients with AN (Figures 1A,B), increasing from 5.1 to 10.0% (FDR = 6.59 × 10^−6^), whereas changes in BN (FDR = 0.739) or other ED (FDR = 0.401) did not reach statistical significance. Compared to the pre-pandemic baseline (Table 1), ED cases did not differ in age at admission (FDR = 0.979) or female preponderance (FDR = 0.925). There was, however, a significant reduction in LOS of *circa* 20 days (FDR = 0.008). There were no significant changes in the total number of diagnoses, the number of co-occurring diagnoses related to somatic or mental health and no significant change in the proportional co-occurrence of either DD or AD in addition to ED during the COVID-19 pandemic compared to the pre-pandemic baseline. There was no change in the ratio of ED cases receiving psychopharmacological intervention. We found no significant changes in the ratio of *de novo* presentations (cases lacking previous admissions two years from index-admission) or the ratio of early re-admissions within six months of the index-admission compared to pre-pandemic times. There was a significant increase from 4.6 to 7.6% in the proportion of hospitalized youth presenting with the three mental disorders ED, DD and AD simultaneously (FDR = 0.003) during the COVID-19 pandemic, also with a significantly shortened LOS (97.5 days vs. 67.6 days, FDR = 0.016).

**Figure 2:**
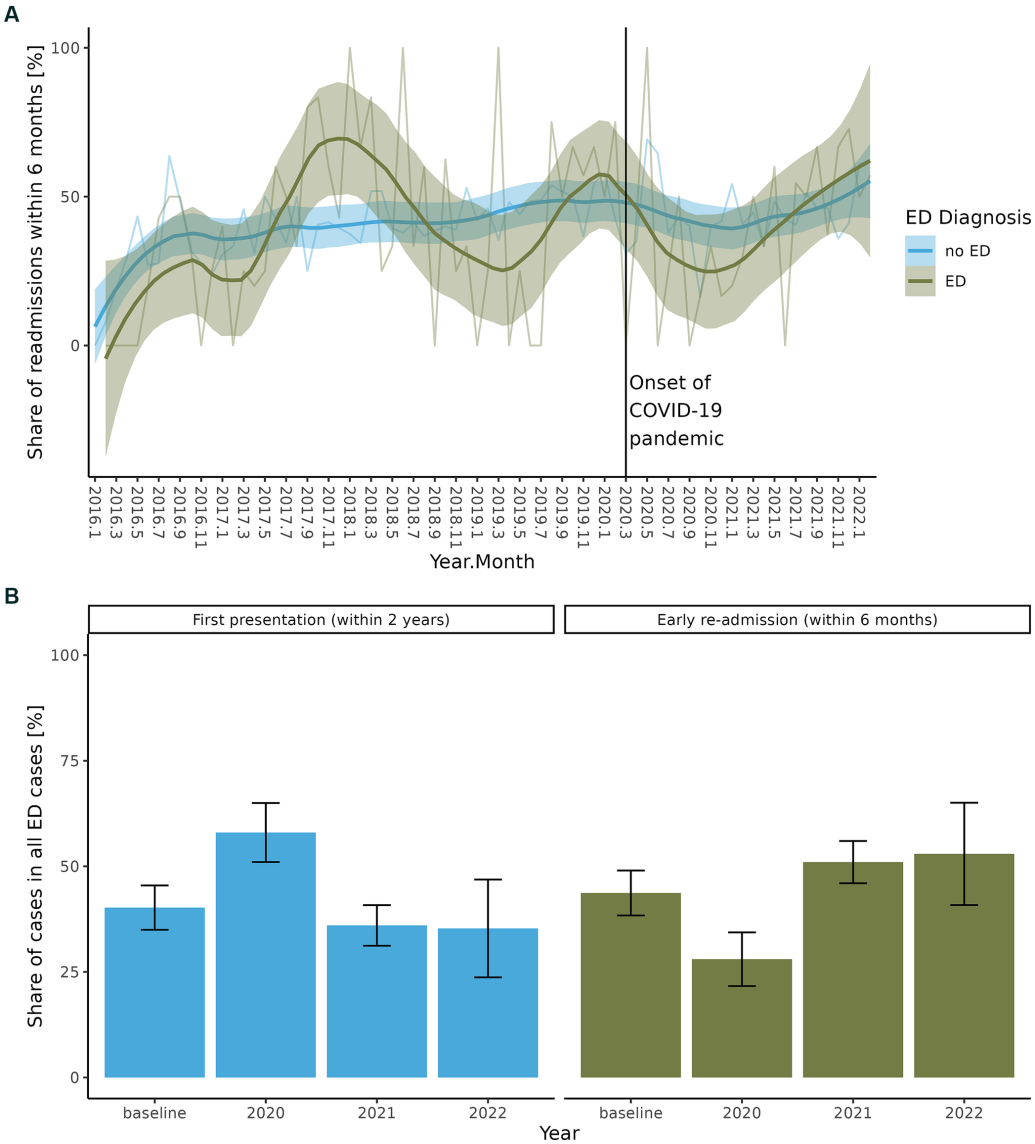
**(A)** Early re-admissions within six months after discharge from inpatient treatment for cases with and without ED during the total study interval 2016–2022. **(B)** Share of de-novo presentations in ED cases and early readmissions in ED cases during each year of the COVID-19 pandemic relative to a pre-pandemic baseline.

For non-ED cases (Table 1), there was no significant change in average age at admission (FDR = 0.446), but there was a significant relative increase in females (54.6% vs. 62.4%, FDR = 0.001). There was a significantly shortened LOS (59.0 days vs. 42.3 days; FDR = 4.06 × 10^−16^) and a significant increase in the total number of diagnoses (3.2 vs. 3.4 diagnoses; FDR = 0.026). The increase in numbers of diagnoses was not caused by changes in diagnoses related to somatic health, but by more comorbid diagnoses related to mental health (1.8 vs. 2.1 diagnoses; FDR = 0.024). Also, relatively more patients with DD were treated during the COVID-19 pandemic (57.3% vs. 68.5%; FDR = 1.05 × 10^−6^) whereas the percentage of AD diagnoses remained the same. Similar to ED cases, there were no significant changes in the proportion of *de novo* admissions or inpatients receiving psychopharmacological intervention in non-ED cases, but there was a significant increase in the ratio of early re-admissions within six months for all 3 years of the COVID-19 pandemic (38.8% vs. 44.1%; FDR = 0.032; Figures 2A,B).

**Figure 3:**
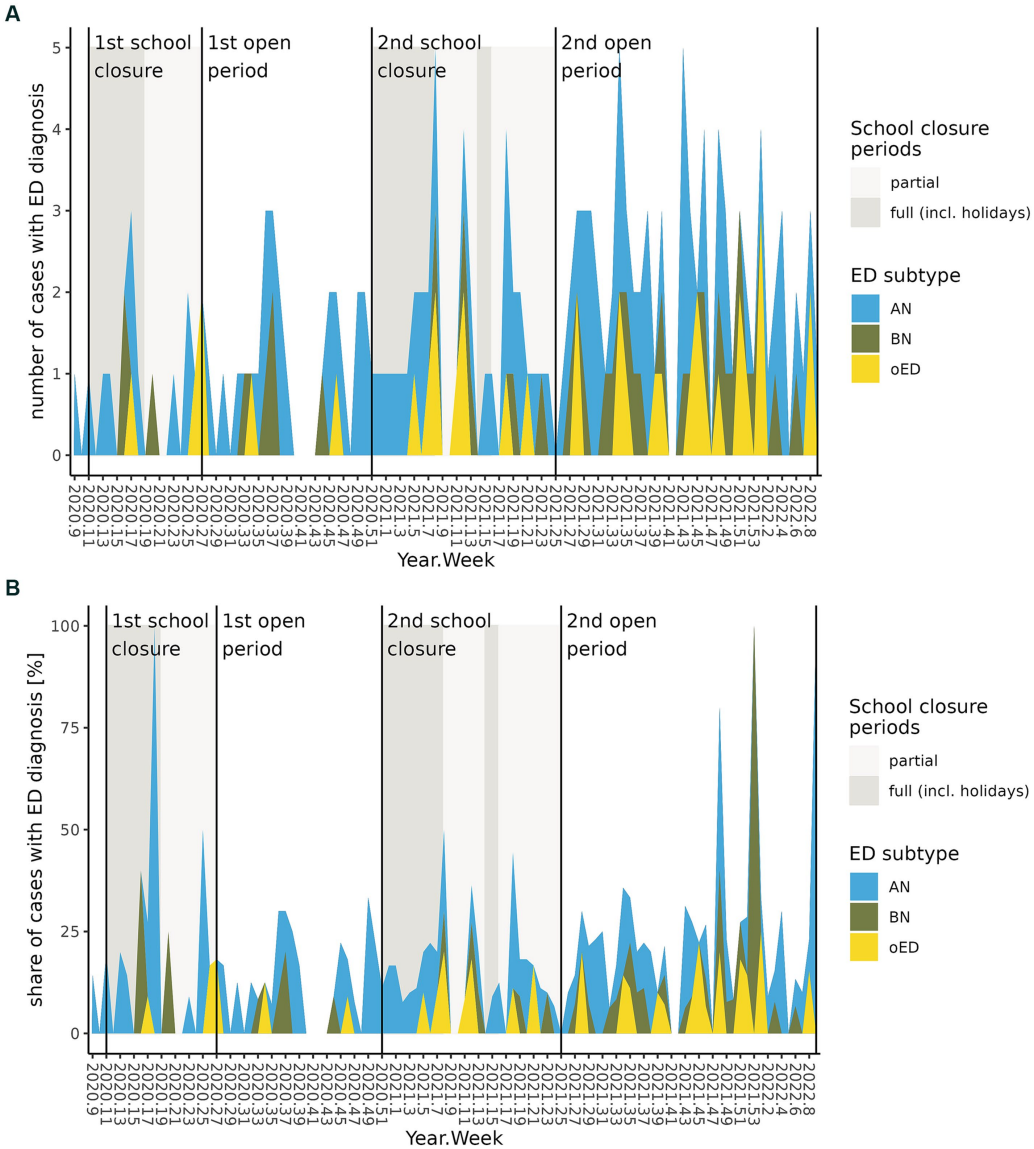
Development of ED inpatient admissions per week during the COVID-19 pandemic interval in combination with Lockdown associated school closures and re-openings. **(A)** Absolute number of ED inpatient admissions. **(B)** ED inpatient admissions as percentage of all inpatient admissions.

We explored the impact of school closures during the two episodes of governmentally mandated lockdown on the ratio of ED admissions (Table 2; Figures 3A,B). There was no influence of school constraints or re-openings on the ratio of ED cases (FDR = 0.572) within a timelag of 6 months. On a descriptive basis, the ratio of ED cases (17.7%) treated at our DCAPPP during episodes with no school constraints within the pandemic interval was larger than the ratio of ED cases during episodes of (partial) school closures (partial closures: 15.5%, full closures: 14.3%). The reopening of schools after the second lockdown episode coincided with the largest increase in the ratio of ED cases (20.3%) and the highest ratio of AN cases (11.0%).

**Table 2:** Statistical analysis of children and youth admitted for inpatient treatment to the Frankfurt DCAPPP during the pandemic interval.

	0th open period	1st school closure	1st open period	2nd school closure	2nd open period	*p* value	FDR
Addmissions total	N = 18	N = 95	N = 199	N = 251	N = 399		
Individuals total	N = 18	N = 94	N = 189	N = 232	N = 347		
Time period (months)	0.39	3.83	5.47	6.33	8.23		
Admissions per month	Npm = 45.00	Npm = 24.79	Npm = 36.39	Npm = 39.64	Npm = 48.488		
Individuals per month	Npm = 45.00	Npm = 24.53	Npm = 34.56	Npm = 36.63	Npm = 42.16		
non-ED cases	88.89% (N = 16, Npm = 40)	84.21% (N = 80, Npm = 20.88)	86.93% (N = 173, Npm = 31.64)	85.26% (N = 214, Npm = 33.8)	79.7% (N = 318, Npm = 38.64)	0.021^a^	0.103
ED cases	11.11% (N = 2, Npm = 5)	15.79% (N = 15, Npm = 3.91)	13.07% (N = 26, Npm = 4.75)	14.74% (N = 37, Npm = 5.84)	20.3% (N = 81, Npm = 9.84)		
AN	11.11% (N = 2, Npm = 5)	8.42% (N = 8, Npm = 2.09)	9.55% (N = 19, Npm = 3.47)	9.16% (N = 23, Npm = 3.63)	11.03% (N = 44, Npm = 5.35)	0.411 ^a^	1
BN	0% (N = 0, Npm = 0)	3.16% (N = 3, Npm = 0.78)	2.51% (N = 5, Npm = 0.91)	1.99% (N = 5, Npm = 0.79)	4.26% (N = 17, Npm = 2.07)	0.164 ^a^	0.804
Other ED	0% (N = 0, Npm = 0)	4.21% (N = 4, Npm = 1.04)	1.01% (N = 2, Npm = 0.37)	3.59% (N = 9, Npm = 1.42)	5.01% (N = 20, Npm = 2.43)	0.047 ^a^	0.231
cases with ED + DD + AD	0% (N = 0, Npm = 0)	7.37% (N = 7, Npm = 1.83)	5.53% (N = 11, Npm = 2.01)	6.77% (N = 17, Npm = 2.68)	9.52% (N = 38, Npm = 4.62)	0.058 ^a^	0.287

a: Spearman-correlation test comparing admissions per month across time bins; N: absolute number; Npm: number per 30 days; SD: standard deviation; ED: Eating disorder; DD: Depressive disorders; AD: Anxiety disorders.

## 4 Discussion

In this study, we investigated the change in absolute and relative admission rates as well as clinical profiles of children and youth with ED following the COVID-19 pandemic at a German, university-hospital based DCAPPP. Based on predominantly Northern American studies, we assumed an increase of AN, accompanied by a younger average age, a higher number of co-occurring mental and somatic disorders, a prolonged LOS and an increase of early re-admissions, all supposedly indicating a higher burden of disease. Additionally, we explored the longitudinal correlation between local school constraints as part of the governmentally mandated lockdown orders and admission rates. We found a significant increase in relative admission rates of children and youth admitted for ED during the COVID-19 pandemic, agreeing with German mandatory health insurance data on out- and inpatients for 2021 (18, 37). In our study, the proportional increase of ED was, however, already apparent at the beginning of the COVID-19 pandemic during the first lockdown in 2020 and persisted well into 2022, also reported by Vyver et al. (21). The separate analysis for ED subgroups proved the relative ED increase to be specifically driven by AN rather than by BN or other ED. Taking our results and internationally available data on incidence, prevalence and hospitalization rates into account (14, 15, 19, 21, 25), it may be concluded, that AN incidence seems to have strongly increased during the COVID-19 pandemic in Western societies. The exact underlying mechanisms have to be determined. Studies addressing possible factors associated with AN incidence increase during the COVID-19 pandemic have found a more intense preoccupation with own’s body image resulting from an increase in social media use and screen time during lockdown episodes while filling the growing amounts of spare time (38, 39).

Looking at the demographic profile of our study population, i.e., age at admission or sex distribution, we found no change during the pandemic, in line with most international studies (33, 40). Whereas the multifaceted psychosocial impact of the COVID-19 pandemic added to the well-specified vulnerabilities and risk factors to develop severe AN, the primary demographic profile did not change, with adolescents still remaining the most vulnerable age group to develop AN. Higher parental supervision of younger children, and intensity of family and peer social interaction may thus be investigated as protective factors in times of environmental stress (1, 41, 42). Considering the relative increase in females regarding ED and non-ED patients, an increased vulnerability of female adolescents to develop or exacerbate mental health disorders in response to environmental stressors can be postulated based on our data, agreeing with international reports (9, 40, 43, 44).

The profile of co-occurring diagnoses of patients with ED in our study population did not change in response to the COVID-19 pandemic with unchanged numbers of co-occurring mental or somatic disorders as well as unchanged rates of co-occurring DD or AD. Accordingly, we did not find an increased need for psychopharmacotherapy in ED patients. Still, we found a significant increase in the admission rate of trifold-affected youth showing ED, DD, and AD, indicating a small group of patients with ED with strongly increased severe mental health problems during COVID-19. Other studies reported a generally increased DD comorbidity in ED inpatients (25, 28) but, similar to our results, no changes in AD comorbidity rates (28, 43, 45).

Whereas our data did not allow for the evaluation of somato-medical information (e.g., BMI-percentiles at admission, laboratory results, cardiac parameters, necessity of tube feeding), the number of co-occurring diagnoses related to somatic health was considered a surrogate for general physical health for the purpose of this study. Contrary to our hypothesis, we did not find an increase in the number of diagnoses related to somatic health in ED patients (COVID-19 related diagnoses excluded), suggesting an unchanged severity of physical deterioration in ED/AN cases. This finding agrees with previous reports (25, 26), where no change in ED symptom severity was found during the first wave of the COVID-19 pandemic. Preexisting or secondary somatic comorbidity did thus not seem to mediate the increase in ED/AN hospitalization rates of youth in response to the COVID-19 pandemic. In addition, the unchanged ED somatic comorbidity seen at our DCAPPP population during COVID-19 indicates an ongoing and prioritized inpatient treatment for children and youth with ED over children and youth with other severe mental disorders, as can also be concluded from the relative decrease in non-ED admission rates during the pandemic. This conclusion is also supported by the increased mental comorbidity rates of children and youth with non-ED diagnoses, and the higher re-admission rate of the non-ED group. International studies investigating patients at pediatric wards found more serious somato-medical outcomes (higher risk for medical instability, higher need for invasive supplementation, more rapid weight loss and ED development, more adverse cardiac parameters) in young ED patients during the COVID-19 pandemic (19, 20, 23, 28). The respective countries are characterized by smaller CAPPP inpatient capacities compared to Germany; thus, within their health care system, an effective triage of ED above other mental disorders may not have been possible, resulting in more chronic and more severely affected ED inpatients and necessitating more emergency admissions to pediatric wards. Still, to rule out selection bias in terms of study setting, analog pandemic data from pediatric wards in Germany might be evaluated. AN cases presenting with greater physical impairment may have been treated more often within the pediatric setting compared to pre-pandemic times. To this end, Kölch et al. (35) recently reported trends of rising AN admissions and an increased LOS of youth with AN in pediatric departments in Germany.

For the Frankfurt DCAPPP we found a significant reduction in LOS for both ED and non-ED cases during the pandemic, in line with Kölch et al. (35) analyzing a similar data set. In contrast, data from two German DCAPPPs from 2020 found LOS unchanged during the early phase of the COVID-19 pandemic (46). The reduction of LOS during the full length of the pandemic at the Frankfurt DCAPPP might at least partly be attributed to opportunity costs of the necessary COVID-19 mitigation measures. Due to intermittently reduced treatment capacity and an increasing ED caseload, triage had to be intensified which may have resulted in shorter LOS. Families’ wishes and/or physicians’ endorsement for a timelier discharge, fearing COVID-19 contagion in a hospital setting especially when little was known about the morbidity of COVID-19 in possibly immunocompromised AN patients, might also have contributed to a shortened LOS. Neither the rate of early re-admissions within 6 months (conceptualized as a surrogate for first time treatment failure), nor the proportion of *de novo* admissions for ED treatment increased during the COVID-19 pandemic. Consequently, the LOS reduction was not associated with a worse treatment outcome for youth suffering from AN, i.e., significant post-hospitalization weight loss leading to early rehospitalization, during the pandemic. Effective acute and intermediate medical and mental health care for relatively more youth suffering from ED/AN compared to pre-pandemic sites was continuously provided by the Frankfurt DCAPPP during the COVID-19 pandemic. Specific characteristics of the German mental health care system for children and youth may have supported this effective care for youth with ED/AN, such as the usual provision of inpatient, day-care, and outpatient care by the same DCAPPP. Due to the overarching structure with one responsible medical director, the system allows flexible triage and specific support for severely affected individuals on an inpatient as well as outpatient basis.

Regarding the comparison group of non-ED cases, we observed an increase in the ratio of females being treated as inpatients. This may indicate a higher vulnerability of female youth to the individual and social stressors induced by the COVID-19 pandemic. This group also suffered from an increased rate of mental health comorbidities and a higher rate of co-occurring depressive disorder. Our results mirror previous reports, suggesting a near doubling of depressive symptoms and a reduction in quality of life in German youth during the COVID-19 pandemic (47, 48). Regarding AD, we found no change in diagnosis rates in the non-ED group. Symptoms of anxiety were found to increase in a general population of youth during the COVID-19 pandemic (8, 48), meta-analytic evidence encompassing primarily adult populations showed an increase in anxiety disorders in 2020 (49). An unchanged ratio of AD in our study may be due to reduced social and academic demands during lockdown-associated remote schooling and more interaction with caregivers during times of increased parental home office.

With regard to the underlying mechanisms, especially of the increased ED and DD inpatient admission rates, it remains unclear as to how specific circumstances during the COVID-19 pandemic impacted on the mental state of vulnerable children and youth, and which specific risk and resilience factors were involved. A growing body of evidence suggests that COVID-19 mitigation strategies showed general and individual effects on vulnerable youth. For example, youth with ED were reported to have suffered higher levels of stress compared to healthy controls during the first episode of social lockdown and school closures (50). We also found an increased AN admission rate already surfacing in the early phases of the COVID-19 pandemic. Regarding general youth mental health, a stronger impact of the COVID-19 pandemic was found during episodes of remote schooling than during episodes of in-classroom-teaching especially on DD and depressive symptoms (1, 2). In our study, in contrast, school closures or re-openings were not longitudinally predictive of an increase in ED or non-ED admission rates, with the relative increase in ED remaining stable throughout the investigated pandemic interval. We thus propose a multifaceted explanation for the heterogenous changes in mental symptoms and admission rates of youth during the COVID-19 pandemic. Given that social media use increased significantly in German youth during the COVID-19 pandemic (51), this secondary effect should be considered in context of changes in youth mental health. Especially for youth with a genetic risk for AN, symptoms may have been triggered by an increase in use of social media during the COVID-19 pandemic (38, 39) and other stressors combined with reduced availability of early professional intervention in some health care systems. Some studies reported a time-lagged increase in ED admission rates during the second year of the COVID-19 pandemic compared to the first year, which we did not observe (50). We are in the process of planning a multicenter study across a number of University Hospital based DCAPPPs in Germany, also including data until 2023, to clarify, if the locally observed increase in ED admission rates occurred nationwide and if such data reflects the clinically observed attenuation of ED admission rates with the subsiding of the COVID-19 pandemic. As the data structure used for this investigation is equally available at other DCAPPPs, this study may serve as a use-case for a multicenter study. To evaluate the impact of the COVID-19 pandemic on youth suffering from ED with lower clinical severity or just emerging symptoms, the study population should be extended to include children and youth with ED in outpatient treatment settings, e.g., patients at outpatient clinics of DCAPPPs, to evaluate the need for early interventions during the COVID-19 pandemic.

### Limitations

Our study comes with a few limitations: Including data from only one urban DCAPPP, sample size and statistical power are limited. A multicenter replication including other DCAPPPs will allow for a larger sample size with increased power to find smaller effects, and investigation of regional differences. Furthermore, biometrical data, such as weight or laboratory results, were not available in our dataset, limiting the analysis of physical illness severity to surrogates, i.e., the number of diagnoses regarding somatic health, without differentiating the etiology of the respective somatic disorders in more detail. Also, the dataset did not include detailed or quantified clinical information on psychiatric symptom severity, (e.g., individual symptom descriptions; psychopathological findings; results from questionnaires), other than expressed by the ICD-10 diagnoses. Thus, our results do not offer insight into severity changes of discrete diagnoses in our inpatient population as a result of the COVID-19 pandemic. Due to the retrospective nature of this study, we could not differentiate whether the increase in DD and AD in ED patients preceded or resulted from ED symptomology or whether this group is characterized by high vulnerability to develop these disorders simultaneously. International studies often reported DSM-5 rather than ICD-10 diagnoses, which might explain some discrepancies of our findings compared to North American studies in particular. We did not include data of pediatric hospitals in this study, thus possibly underreporting emergency admissions of ED youth due to somatic complications. Still, in the German system, the vast majority of youth with ED is treated in DCAPPPs, where both, physical needs and complications of AN as well as psychiatric care can be delivered simultaneously. Thus, our study likely covered the majority of ED youth living in Frankfurt.

### Conclusion

To our knowledge, this study is the first to analyze routine hospital data of admission rates and clinical profiles of children and youth suffering from severe ED, including ED-subgroups, and compare these with non-ED patients from a large German city, studying the effect of the COVID-19 pandemic and its mitigation measure, i.e., all school closure episodes, until spring 2022. Throughout the COVID-19 pandemic but independent of both episodes of school closures, we found a higher burden of mental disorders in youth hospitalized for reasons other than ED, and an increased ratio of youth hospitalized for ED treatment, mainly driven by female youth suffering from AN, prompting a shortened LOS as well as a stricter triage necessity for other non-ED admissions. These findings pinpoint the need for adequate mental health treatment capacities during environmental crises, and continuous strengthening of DCAPP services in Germany.

## Data availability statement

The data analyzed in this study is subject to the following licenses/restrictions: The data presented in this study underly local policies and are not available on request from the corresponding author. The respective data can be requested through the “Forschungsdatenportal für Gesundheit” (FDPG): https://www.forschen-fuer-gesundheit.de/. The analysis code is available as git-repository: https://github.com/KJPMolgenLab/coverCHILD_ED_UKF_2308. Requests to access these datasets should be directed to ann-sophieluise.silber@kgu.de.

## Ethics statement

The studies involving humans were approved by the medical faculty’s Ethics Board of Goethe University Frankfurt (13/06/2022, number 2022-804). The studies were conducted in accordance with the local legislation and institutional requirements. Written informed consent for participation was not required from the participants or the participants' legal guardians/next of kin in accordance with the national legislation and institutional requirements.

## Author contributions

A-SS: Writing – original draft. SP: Data curation, Formal analysis, Investigation, Methodology, Software, Visualization, Writing – review & editing. AK: Formal analysis, Writing – review & editing. SA: Methodology, Writing – review & editing. DK: Data curation, Writing – review & editing. MS: Data curation, Writing – review & editing. HS: Data curation, Writing – review & editing. AC: Formal analysis, Methodology, Project administration, Supervision, Visualization, Writing – review & editing. CF: Conceptualization, Project administration, Supervision, Writing – review & editing.

## References

[1] Saulle R De SM Bena A Capra P Culasso M Davoli M. Chiusura della scuola e salute mentale, benessere e comportamenti correlati alla salute in bambini e adolescenti durante la seconda ondata di COVID-19: una revisione sistematica della letteratura. Epidemiol Prev. (2022) 46:333–52. doi: 10.19191/EP22.5-6.A542.089, PMID: 36384255

[2] Viner R Russell S Saulle R Croker H Stansfield C Packer J. School closures during social lockdown and mental health, health behaviors, and well-being among children and adolescents during the first COVID-19 wave: a systematic review. JAMA Pediatr. (2022) 176:400–9. doi: 10.1001/jamapediatrics.2021.5840 35040870

[3] Chaabane S Doraiswamy S Chaabna K Mamtani R Cheema S. The impact of COVID-19 school closure on child and adolescent health: a rapid systematic review. Children (Basel). (2021) 8:8050415. doi: 10.3390/children8050415 PMC8159143 34069468

[4] Kauhanen L Wan Mohd Yunus WMA Lempinen L Peltonen K Gyllenberg D Mishina K. A systematic review of the mental health changes of children and young people before and during the COVID-19 pandemic. Eur Child Adolesc Psychiatry. (2022) 32:995–1013. doi: 10.1007/s00787-022-02060-0 35962147 PMC9373888

[5] Wang S Chen L Ran H Che Y Fang D Sun H. Depression and anxiety among children and adolescents pre and post COVID-19: a comparative meta-analysis. Front Psych. (2022) 13:917552. doi: 10.3389/fpsyt.2022.917552, PMID: 35990058 PMC9381924

[6] Radhakrishnan L Leeb RT Bitsko RH Carey K Gates A Holland KM. Pediatric emergency department visits associated with mental health conditions before and during the COVID-19 pandemic - United States, January 2019-January 2022. MMWR Morb Mortal Wkly Rep. (2022) 71:319–24. doi: 10.15585/mmwr.mm7108e2, PMID: 35202358

[7] Shankar LG Habich M Rosenman M Arzu J Lales G Hoffmann JA. Mental health emergency department visits by children before and during the COVID-19 pandemic. Acad Pediatr. (2022) 22:1127–32. doi: 10.1016/j.acap.2022.05.022, PMID: 35667622 PMC9164513

[8] Ravens-Sieberer U Erhart M Devine J Gilbert M Reiss F Barkmann C. Child and adolescent mental health during the COVID-19 pandemic: results of the three-wave longitudinal COPSY study. J Adolesc Health. (2022) 71:570–8. doi: 10.1016/j.jadohealth.2022.06.022, PMID: 35989235 PMC9386895

[9] Meherali S Punjani N Louie-Poon S Abdul Rahim K Das JK Salam RA. Mental health of children and adolescents amidst COVID-19 and past pandemics: a rapid systematic review. Int J Environ Res Public Health. (2021) 18:18073432. doi: 10.3390/ijerph18073432, PMID: 33810225 PMC8038056

[10] Westmoreland P Krantz MJ Mehler PS. Medical complications of anorexia nervosa and bulimia. Am J Med. (2016) 129:30–7. doi: 10.1016/j.amjmed.2015.06.031 26169883

[11] Attia E. Anorexia nervosa: current status and future directions. Annu Rev Med. (2010) 61:425–35. doi: 10.1146/annurev.med.050208.200745, PMID: 19719398

[12] Khraisat BR Al-Jeady AM Alqatawneh DA Toubasi AA AlRyalat SA. The prevalence of mental health outcomes among eating disorder patients during the COVID-19 pandemic: a meta-analysis. Clin Nutr ESPEN. (2022) 48:141–7. doi: 10.1016/j.clnesp.2022.01.033, PMID: 35331484 PMC8810264

[13] Haghshomar M Shobeiri P Brand S Rossell SL Akhavan Malayeri A Rezaei N. Changes of symptoms of eating disorders (ED) and their related psychological health issues during the COVID-19 pandemic: a systematic review and meta-analysis. J Eat Disord. (2022) 10:51. doi: 10.1186/s40337-022-00550-9, PMID: 35418108 PMC9006500

[14] Devoe D J Han A Anderson A Katzman DK Patten SB Soumbasis A. The impact of the COVID-19 pandemic on eating disorders: a systematic review. Int J Eat Disord. (2022) 56:5–25. doi: 10.1002/eat.23704 35384016 PMC9087369

[15] Linardon J Messer M Rodgers RF Fuller-Tyszkiewicz M. A systematic scoping review of research on COVID-19 impacts on eating disorders: a critical appraisal of the evidence and recommendations for the field. Int J Eat Disord. (2022) 55:3–38. doi: 10.1002/eat.23640, PMID: 34773665 PMC8646470

[16] Gao Y Bagheri N Furuya-Kanamori L. Has the COVID-19 pandemic lockdown worsened eating disorders symptoms among patients with eating disorders? A systematic review Z Gesundh Wiss. (2022) 30:2743–52. doi: 10.1007/s10389-022-01704-4, PMID: 35369670 PMC8961480

[17] Grilo CM Udo T. Examining the significance of age of onset in persons with lifetime anorexia nervosa: comparing child, adolescent, and emerging adult onsets in nationally representative U.S. study. Int J Eat Disord. (2021) 54:1632–40. doi: 10.1002/eat.23580, PMID: 34263464 PMC8416938

[18] Herpertz-Dahlmann B Dahmen B. Children in need-diagnostics, epidemiology, treatment and outcome of early onset anorexia nervosa. Nutrients. (2019) 11:11081932. doi: 10.3390/nu11081932, PMID: 31426409 PMC6722835

[19] Agostino H Burstein B Moubayed D Taddeo D Grady R Vyver E. Trends in the incidence of new-onset anorexia nervosa and atypical anorexia nervosa among youth during the COVID-19 pandemic in Canada. JAMA Netw Open. (2021) 4:e2137395. doi: 10.1001/jamanetworkopen.2021.37395, PMID: 34874405 PMC8652595

[20] Spettigue W Obeid N Erbach M Feder S Finner N Harrison ME. The impact of COVID-19 on adolescents with eating disorders: a cohort study. J Eat Disord. (2021) 9:65. doi: 10.1186/s40337-021-00419-3 34088342 PMC8176274

[21] Vyver E Han AX Dimitropoulos G Patten SB Devoe DJ Marcoux-Louie G. The COVID-19 pandemic and Canadian pediatric tertiary care hospitalizations for anorexia nervosa. J Adolesc Health. (2022) 72:344–51. doi: 10.1016/j.jadohealth.2022.07.003 36202680 PMC9529357

[22] Matthews A Kramer RA Peterson CM Mitan L. Higher admission and rapid readmission rates among medically hospitalized youth with anorexia nervosa/atypical anorexia nervosa during COVID-19. Eat Behav. (2021) 43:101573. doi: 10.1016/j.eatbeh.2021.101573, PMID: 34619464 PMC8490008

[23] Feldman MA King CK Vitale S Denhardt B Stroup S Reese J. The impact of COVID-19 on adolescents with eating disorders: increased need for medical stabilization and decreased access to care. Int J Eat Disord. (2022) 56:257–62. doi: 10.1002/eat.23788 35906993 PMC9353287

[24] Datta N van Wye E Citron K Matheson B Lock JD. The COVID-19 pandemic and youth with anorexia nervosa: a retrospective comparative cohort design. Int J Eat Disord. (2023) 56:263–8. doi: 10.1002/eat.23817, PMID: 36125016 PMC9538435

[25] Springall G Cheung M Sawyer SM Yeo M. Impact of the coronavirus pandemic on anorexia nervosa and atypical anorexia nervosa presentations to an Australian tertiary paediatric hospital. J Paediatr Child Health. (2022) 58:491–6. doi: 10.1111/jpc.15755 34570958 PMC8661708

[26] Goldberg L Ziv A Vardi Y Hadas S Zuabi T Yeshareem L. The effect of COVID-19 pandemic on hospitalizations and disease characteristics of adolescents with anorexia nervosa. Eur J Pediatr. (2022) 181:1767–71. doi: 10.1007/s00431-021-04350-2, PMID: 34981183 PMC8722655

[27] Spina G Roversi M Marchili MR Raucci U Fini F Mirra G. Psychiatric comorbidities and dehydration are more common in children admitted to the emergency department for eating disorders in the COVID-19 era. Eat Weight Disord. (2022) 27:2473–80. doi: 10.1007/s40519-022-01386-7, PMID: 35294772 PMC8925290

[28] Girardi M Assalone C Maines E Genovese A Naselli A Nai Fovino L. Disease characteristics and psychiatric comorbidities in adolescents with anorexia nervosa hospitalized during COVID-19 pandemic. Front Biosci (Schol Ed). (2022) 14:28. doi: 10.31083/j.fbs1404028, PMID: 36575838

[29] Surén P Skirbekk AB Torgersen L Bang L Godøy A Hart RK. Eating disorder diagnoses in children and adolescents in Norway before vs during the COVID-19 pandemic. JAMA Netw Open. (2022) 5:e2222079. doi: 10.1001/jamanetworkopen.2022.22079, PMID: 35816316 PMC9280394

[30] Kersten JM van Veen M van Houten MA Wieringa J Noordzij JG Bekhof J. Adverse effect of lockdowns during the COVID-19 pandemic: increased incidence of pediatric crisis admissions due to eating disorders and adolescent intoxications. Eur J Pediatr. (2023) 182:1137–42. doi: 10.1007/s00431-022-04773-5 36598566 PMC9811038

[31] Gilsbach S Herpertz-Dahlmann B Konrad K. Psychological impact of the COVID-19 pandemic on children and adolescents with and without mental disorders. Front Public Health. (2021) 9:679041. doi: 10.3389/fpubh.2021.679041, PMID: 34805060 PMC8602182

[32] Muth L Leven K-H Moll G Kratz O Horndasch S. Effects of the COVID-19 restrictions on eating behaviour and eating disorder symptomology in female adolescents. Int J Environ Res Public Health. (2022) 19:19148480. doi: 10.3390/ijerph19148480, PMID: 35886334 PMC9325224

[33] Shum M Moreno C Kamody R McCollum S Shabanova V Loyal J. The evolving needs of children hospitalized for eating disorders during the COVID-19 pandemic. Hosp Pediatr. (2022) 12:696–702. doi: 10.1542/hpeds.2022-006545, PMID: 35815415

[34] Allgaier K Schneider PS Buck S Reusch PA Hagmann D Barth GM. Kinder- und jugendpsychiatrische Notfälle während der zweiten Welle der SARS-CoV2-19-Pandemie. Z Kinder Jugendpsychiatr Psychother. (2022) 50:275–85. doi: 10.1024/1422-4917/a000858, PMID: 35225657

[35] Kölch MG Reis O Ulbrich L Schepker R. COVID-19 und psychische Störungen bei Minderjährigen: Veränderungen der Behandlungen nach der Krankenhausstatistik. Z Kinder Jugendpsychiatr Psychother. (2023) 51:295–309. doi: 10.1024/1422-4917/a000935, PMID: 37166813

[36] Steinmetz H Batzdorfer V Scherhag J Bosnjak M. The ZPID Lockdown Measures Dataset for Germany [Data set]. PsychArchives. (2022). doi: 10.23668/PSYCHARCHIVES.6676

[37] Witte J. Kinder- und Jugendreport. Gesundheitsversorgung von Kindern und Jugendlichen in Deutschland Schwerpunkt: Suchterkrankungen. Beiträge zur Gesundheitsökonomie und Versorgungsforschung (Band 36). (2021). Available at: https://www.dak.de/dak/download/report-2519092.pdf

[38] Gobin KC Mills JS McComb SE. The effects of the COVID-19 pandemic lockdown on eating, body image, and social media habits among women with and without symptoms of orthorexia nervosa. Front Psychol. (2021) 12:716998. doi: 10.3389/fpsyg.2021.716998, PMID: 34975611 PMC8714632

[39] Vall-Roqué H Andrés A Saldaña C. The impact of COVID-19 lockdown on social network sites use, body image disturbances and self-esteem among adolescent and young women. Prog Neuro-Psychopharmacol Biol Psychiatry. (2021) 110:110293. doi: 10.1016/j.pnpbp.2021.110293, PMID: 33662532 PMC8569938

[40] Gutiérrez-Sacristán A Serret-Larmande A Hutch MR Sáez C Aronow BJ Bhatnagar S. Hospitalizations Associated With Mental Health Conditions Among Adolescents in the US and France During the COVID-19 Pandemic. JAMA Netw Open. (2022) 5:e2246548. doi: 10.1001/jamanetworkopen.2022.46548, PMID: 36512353 PMC9856226

[41] Bobo E Lin L Acquaviva E Caci H Franc N Gamon L. Comment les enfants et adolescents avec le trouble déficit d’attention/hyperactivité (TDAH) vivent-ils le confinement durant la pandémie COVID-19? Encephale. (2020) 46:S85–92. doi: 10.1016/j.encep.2020.05.011, PMID: 32522407 PMC7276130

[42] Samuels A Avital-Magen A Schusheim G. The Effects of the First Phase of the Covid-19 Pandemic on the Mental Health of Children and Adolescents With preexisting Psychiatric Conditions. Clin Pediatr (Phila). (2023) 99228221150157 62:1008–17. doi: 10.1177/00099228221150157, PMID: 36726020 PMC9899673

[43] Schreyer CC Vanzhula IA Guarda AS. Evaluating the impact of COVID-19 on severity at admission and response to inpatient treatment for adult and adolescent patients with eating disorders. Int J Eat Disord. (2023) 56:182–91. doi: 10.1002/eat.23855, PMID: 36394170

[44] Millner AJ Zuromski KL Joyce VW Kelly F Richards C Buonopane RJ. Increased severity of mental health symptoms among adolescent inpatients during COVID-19. Gen Hosp Psychiatry. (2022) 77:77–9. doi: 10.1016/j.genhosppsych.2022.04.004, PMID: 35569321 PMC8996442

[45] Güzel Â Mutlu NL Molendijk M. COVID-19-related changes in eating disorder pathology, emotional and binge eating and need for care: a systematic review with frequentist and Bayesian meta-analyses. Eat Weight Disord. (2023) 28:19. 36805344 10.1007/s40519-023-01547-2 PMC9941242

[46] Gilsbach S Plana MT Castro-Fornieles J Gatta M Karlsson GP Flamarique I. Increase in admission rates and symptom severity of childhood and adolescent anorexia nervosa in Europe during the COVID-19 pandemic: data from specialized eating disorder units in different European countries. Child Adolesc Psychiatry Ment Health. (2022) 16:46. 35725621 10.1186/s13034-022-00482-x PMC9208345

[47] Racine N McArthur BA Cooke JE Eirich R Zhu J Madigan S. Global Prevalence of Depressive and Anxiety Symptoms in Children and Adolescents During COVID-19: A Meta-analysis. JAMA Pediatr. (2021) 175:1142–50. doi: 10.1001/jamapediatrics.2021.2482, PMID: 34369987 PMC8353576

[48] Kostev K Weber K Riedel-Heller S, Vultée C von, Bohlken J. Increase in depression and anxiety disorder diagnoses during the COVID-19 pandemic in children and adolescents followed in pediatric practices in Germany. Eur Child Adolesc Psychiatry (2021):873–879, 32, doi: 10.1007/s00787-021-01924-1 34825964 PMC8619647

[49] Santabárbara J Lasheras I Lipnicki DM Bueno-Notivol J Pérez-Moreno M López-Antón R. Prevalence of anxiety in the COVID-19 pandemic: An updated meta-analysis of community-based studies. Prog Neuropsychopharmacol Biol Psychiatry. (2021) 109:110207 33338558 10.1016/j.pnpbp.2020.110207 PMC7834650

[50] Sideli L Lo Coco G Bonfanti RC Borsarini B Fortunato L Sechi C. Effects of COVID-19 lockdown on eating disorders and obesity: A systematic review and meta-analysis. Eur Eat Disord Rev. (2021) 29:826–41. doi: 10.1002/erv.2861, PMID: 34460991 PMC8652707

[51] Bodanowitz J. Mediensucht 2020 – Gaming und Social Media in Zeiten von Corona: DAK-Längsschnittstudie: Befragung von Kindern, Jugendlichen (12 – 17 Jahre) und deren Eltern. DAK Gesundheit. (2020) Available at: https://caas.content.dak.de/caas/v1/media/12654/data/e364341b499ec01105a44cdd5eed6f97/dak-studie-gaming-social-media-und-corona.pdf

